# 'Visual’ parsing can be taught quickly without visual experience during critical periods

**DOI:** 10.1038/srep15359

**Published:** 2015-10-20

**Authors:** Lior Reich, Amir Amedi

**Affiliations:** 1 Department of Medical Neurobiology, The Institute for Medical Research Israel-Canada, Faculty of Medicine, The Hebrew University of Jerusalem, Jerusalem 91220, Israel; 2 The Edmond and Lily Safra Center for Brain Sciences (ELSC), The Hebrew University of Jerusalem, Jerusalem 91220, Israel

## Abstract

Cases of invasive sight-restoration in congenital blind adults demonstrated that acquiring visual abilities is extremely challenging, presumably because visual-experience during critical-periods is crucial for learning visual-unique concepts (e.g. size constancy). Visual rehabilitation can also be achieved using sensory-substitution-devices (SSDs) which convey visual information non-invasively through sounds. We tested whether one critical concept – visual parsing, which is highly-impaired in sight-restored patients – can be learned using SSD. To this end, congenitally blind adults participated in a unique, relatively short (~70 hours), SSD-‘vision’ training. Following this, participants successfully parsed 2D and 3D visual objects. Control individuals naïve to SSDs demonstrated that while some aspects of parsing with SSD are intuitive, the blind’s success could not be attributed to auditory processing alone. Furthermore, we had a unique opportunity to compare the SSD-users’ abilities to those reported for sight-restored patients who performed similar tasks visually, and who had months of eyesight. Intriguingly, the SSD-users outperformed the patients on most criteria tested. These suggest that with adequate training and technologies, key high-order visual features can be quickly acquired in adulthood, and lack of visual-experience during critical-periods can be somewhat compensated for. Practically, these highlight the potential of SSDs as standalone-aids or combined with invasive restoration approaches.

39,000,000 people worldwide are blind, constituting a major clinical challenge to develop effective visual rehabilitation techniques. The most straightforward clinical approach is to surgically correct the function of the eyes’ non-neural components (e.g. by removing cataracts, which are the major cause of blindness in developing countries due to low treatment accessibility, or by corneal transplantation). Such treatments result in nearly full resolution of visual input. However they are only applicable to specific causes and stages of vision loss. To treat other blindness etiologies which damage the retina, visual prostheses^1^,^2^,^3^,^4^ are being developed (for current visual performance using prostheses see^1^,^2^,^5^,^6^,^7^,^8^). This promising field is growing extremely fast, and involves massive research, as well as engineering and economic efforts.

However, even if full resolution of visual input is delivered to the brain (as in cataract removal; but this is far from being the case with current prostheses, which provide very low-resolution information), the acquisition of higher visual function in adulthood is still very challenging, even after weeks, months or years of rich post-surgery visual experience. Thus, reports on individuals^9^,^10^,^11^,^12^,^13^,^14^,^15^ who had limited or no visual-experience during development and medically regained fairly complete visual input in adulthood have found profound deficits in various visual skills. While some functions (e.g. motion detection, basic form recognition) recovered relatively fast, many others (e.g. 3D perception, object and face recognition, interpretation of transparency and perspective cues) were massively impaired and recovered slowly (if at all). It seems as if the regained visual input have been provided to a brain that was wholly unpracticed at analyzing and interpreting it, and the visual-experience acquired at this stage may have come too late or too little. This is commonly hypothesized to result from the absence of natural visual information during critical (or sensitive) periods, a notion first introduced by Hubel and Wiesel^16^,^17^ who showed in animal models that even short visual deprivation durations during early developmental stages may irreversibly damage visual perception at older ages. Notably, an even short period of congenital blindness in humans, although treated in early childhood, can lead to some persistent (though much less dramatic) functional deficits^18^,^19^,^20^,^21^.

One highly important task consistently reported to be impaired following sight restoration in adulthood^13^ is visual parsing; i.e., the ability to segregate a visual scene into distinct, complete objects. Consider for instance a typical office desk, with a computer screen, a keyboard and some stationery on it. When looking at the scene you do not perceive a messy collection of areas of different hues, luminance levels, textures and contours, but rather see separate meaningful objects. While this parsing task seems trivial to the normally-developed sighted, it is very complex and demanding, sometimes almost impossible, for a person with limited visual experience as it requires interpreting the visual input based on previous knowledge and visual concepts which have no intuitive parallel in other sensory modalities (e.g. shadow, transparency)^22^. It is worth noting that visual parsing is extremely difficult even for most computer-vision algorithms, as they are based on basic image-driven features such as continuity of grey-level and bounding contours^23^ and lack higher-order feedback input, which has an important role in object perception^24^.

An elegant study by Ostrovsky and colleagues^13^ showed that individuals who had their sight restored medically (by cataract removal or refractive correction^13^) performed very poorly in visual parsing of much simpler images than the scene described above: when attempting to parse an image, they made judgments based only on color, closed loops, luminance levels and motion cues, and did not apply any higher-order visual interpretation, and thus over-fragmented the image. For instance, they misinterpreted a 3D cube to be three different patches in different grayscale levels.

Here we took advantage of a unique structured-training program that was developed and has been perfected in our lab for the last 7 years, which enables the blind to ‘see’ using another class of visual rehabilitation approaches – non-invasive sensory substitution devices (SSDs) – and tested whether training the adult brain could help acquire this key function.

Visual-to-auditory SSDs (Supp. Fig. 1A) transform visual images into sound representations (‘soundscapes’), while preserving the image’s spatial topography (Supp. Fig. 1B), thus theoretically enabling the blind to ‘see’ using their ears in a cheap and easily accessible manner. Whether these SSDs are useful and successful for visual rehabilitation is still an open question, but one that has elicited growing interest in recent years. Although there is accumulating evidence demonstrating functional abilities in various ‘visual’ tasks using SSDs^25^,^26^,^27^,^28^,^29^,^30^, no group has directly tested ‘visual’ parsing - one of the most basic functions which is fundamental for recognizing objects and interacting with them, and thus for the practical use of SSDs. We are also not aware of any formal organized programs to teach SSD usage, which is one of the main limitations in their adoption.

The main aims of the current study were thus to: 1) test whether the concept of ‘visual’ parsing (and the required underlying visual knowledge, such as understanding transparency) can be acquired in adulthood by the congenitally blind who lack any visual experience, and whether it can be implemented practically using the vOICe SSD^31^ after limited training; 2) take advantage of a unique opportunity to compare, at least to some extent, the parsing abilities of the SSD-users to those reported^13^ for sight-restored individuals. Specifically, can the use of SSD to perceive ‘visual’ information help overcome some of the challenges observed in the patients?

As an additional related question, given the topographical nature of the vOICe SSD, we assessed to what extent the parsing task could be performed intuitively without any training by sighted individuals.

## Results

All blind participants were enrolled in a novel unique structured-training program in which they learned how to extract and interpret high-resolution visual information from the complex soundscapes generated by the vOICe SSD (Supp. Fig. 1; see Methods for full details). Each subject underwent ~70 hours of one-on-one training, in 2-hour weekly sessions. The program was composed of two main components: structured 2D training in lab-settings, and live-view training in more natural settings. During the 2D stage participants were taught how to process the soundscapes of 2D static images from various visual categories (Supp. Fig. 1D). During each training trial, the participants heard a soundscape and were asked to describe the image, pay attention to both the location and the shapes of all elements in the image, as well as integrate the details into meaningful wholes. Additionally, more general visual principles such as the conversion of 3D objects to 2D images (and back) were demonstrated. Training was conducted using guiding questions, verbal explanations and tangible-images feedback (see Supp. Fig. 1E). In the initial stages of training the participants were also asked to draw, by engraving, the ‘visual’ mental image constructed in their mind’s eye. After this structured-training, participants could indicate which category a soundscape represented^29^, and identify multiple features enabling a differentiation between objects in the same category. During live-viewing training, participants used a mobile kit of the vOICe (Supp. Fig. 1C) to acquire on-line dynamic images and actively sense the environment, thus making the transformation from perception to action. Visual knowledge and skills were also introduced at this stage. E.g., the change in the size of a seen object with distance was counter-intuitive for the participants, since this is not the case when judging an object’s size and distance by touch, and we had to explicitly explain it and intensively practice its implications. Similarly, they practiced head-“eye”-hand coordination, orienting their heads (and the sunglasses supporting the camera) to the objects at hand, etc.

**Figure 1:**
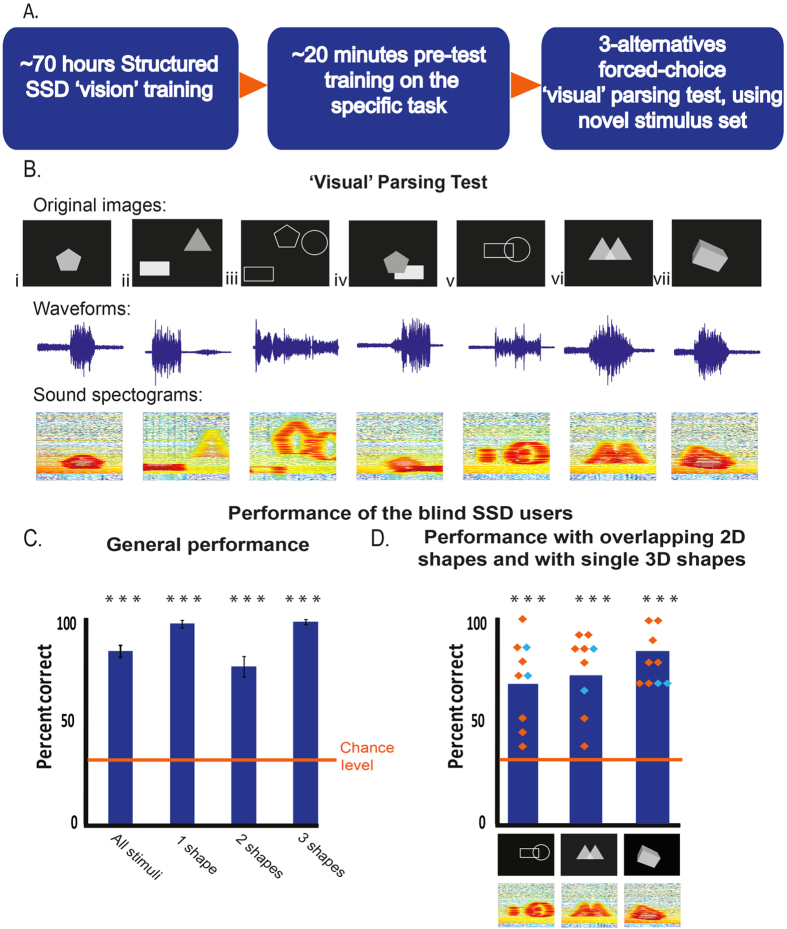
Success of congenitally blind users of the vOICe SSD on the ‘visual’ parsing test. (**A**) Skills and principles required for ‘visual’ parsing were not directly taught during the ~70 hours training, but rather were introduced very briefly (~20 minutes) in a pre-test training session. Different stimuli were used in the training and test phases. (**B**) Types of stimuli presented during the ‘visual’ parsing test: a) 1, 2 or 3 non-overlapping shapes (filled opaque or line drawing; see examples in i-iii); b) 2 overlapping shapes (filled opaque, line drawing or filled transparent; iv-vi); c) a single 3D shape (vii). Below each stimulus is the waveform and spectrogram (demonstrating that visual shape is preserved in the sound) of its soundscape. (**C**) The SSD-users performed significantly above chance in indicating the number of 2D shapes. Error bars represent standard error of the mean. (**D**) The SSD-users performed significantly above chance in parsing overlapping 2D objects and single 3D shapes. The average performance of the 7 congenitally fully blind individuals is depicted by blue bars, the performance of individual congenitally fully blind SSD-users in orange diamonds, the performance of individual SSD-users who had very limited visual experience in cyan diamonds. *** denotes p < 0.0006.

Importantly, the skills tested here were not directly taught during this general structured-training program, but were only introduced in a short pre-test training session (which included completely different stimuli than those used in the test; Fig. 1A).

In the ‘visual’ parsing test, 7 congenitally fully blind adults were presented with soundscapes of images containing 1, 2 or 3 shapes and were requested to indicate the number of objects. Specifically, there were a few types of stimuli: a) 1, 2 or 3 non-overlapping shapes (filled opaque, line drawings or filled transparent; see examples in Fig. 1B i-iii); b) 2 overlapping shapes (filled opaque, line drawings or filled transparent; Fig. 1B iv–vi); c) a single 3D shape (Fig. 1B vii).

The total success rate of the SSD-users group was 84.1% ± s.d 7.6, with a performance of 97.1% ± 4.9, 76.7% ± 13.2 and 98.1% ± 3.3 for stimuli containing 1, 2 or 3 2D shapes, respectively (Fig. 1C; for detailed performance in each stimulus type see Supp. Fig. 2). All success rates were highly significant above chance-level (33.3% on a 3-alternative-forced choice; p < 0.0006 (n = 7) for all comparisons, as assessed by a Wilcoxon rank sum test; importantly, this is the lowest p-value possible in this non-parametric test, given the number of subjects. All p-values reported here were corrected for multiple comparisons using the most conservative Bonferroni correction).

In order to account not only for the participants’ success rate but also to the errors committed we further calculated the d’ sensitivity measure. Averaged d’ was 3.6 ± 1.2, 2.5 ± 0.4 and 5.6 ± 2 for responding “1”, “2” or “3”, respectively. The full data matrix of the participants’ responses is presented in Supp. Table 1.

The average reaction time per stimulus was 7.4 ± 3.2 seconds, i.e. 3.7 repetitions of the stimuli (since the scanning rate was 2 seconds per image). No significant correlation (r^2^ = 0.366) was found between participants’ performance and reaction time (Supp. Fig. 3). We next looked specifically at our subjects’ ability to identify two overlapping shapes as two distinct objects, a highly impaired ability in sight-restored individuals even after weeks to months of visual experience^13^. The SSD-users performed significantly above chance (73% ± 17.5; p < 0.0006), regardless of whether the overlapping shapes were presented as line drawings or as transparent shapes (68.6% ± 22.3 and 76.2% ± 21 respectively, p < 0.0006, Fig. 1D).

Since sight-restored individuals have been reported to successfully parse overlapping shapes when these were in different colors^13^, we also tested our subjects’ ability to parse two overlapping opaque shapes of different luminance levels, which is the closest parallel to color in the grayscale-only conversion of the vOICe. In this case as well, the SSD-users were very successful (72.4% ± 16.5 correct; Supp. Fig. 2).

In addition to the group of 7 congenitally fully blind individuals, we also tested 2 subjects who had some very limited visual experience. FN has faint light (but not form) perception, and HBH had some vision in one eye during her first year of life (Table 1). These 2 subjects performed similarly to the group (Fig. 1C, represented by cyan diamonds; 82.1% and 86.3% total performance, 77.8% and 75.6% parsing the two overlapping shapes for FN and HBH, respectively).

Next, we assessed whether the ‘visual’ parsing capacity of the blind using SSD extends to 3D objects, by testing their ability to perceive 3D shapes as single entities, despite the fact that the shape is made up of several facets with different luminance levels. The congenitally fully blind SSD-users performed very well (Fig. 1D; 84.3% ± 12.7; p < 0.0006). FN and HBH were also successful and both had 70% success.

**Figure 2:**
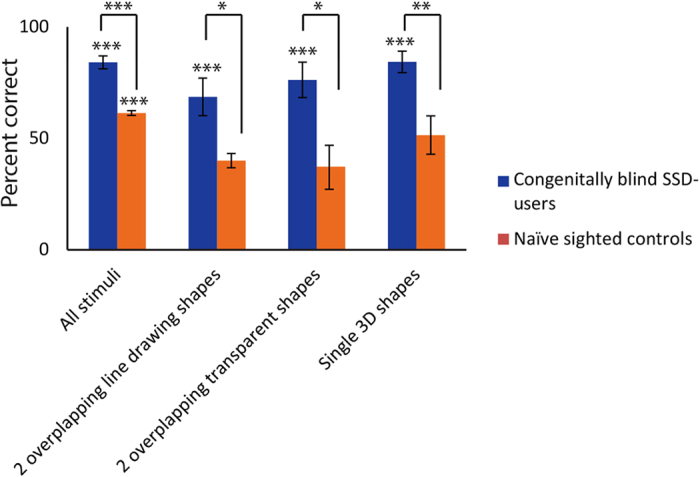
Comparison of performance between the highly-trained congenitally blind SSD-users and a group of sighted controls totally naïve to SSD. The group of 7 congenitally and fully blind SSD-users (blue bars) performed significantly better than a group of 7 age- and gender-matched naïve sighted individuals (orange bars). Error bars represent standard error of the mean. *** denotes p < 0.0006. ** denotes p < 0.005. * denotes p < 0.05.

In order to verify that the blind SSD users’ ‘visual’ parsing ability could not be attributed to auditory processing alone, and to assess what level of parsing can be achieved without any training, 7 sighted controls, matched to the group of 7 congenitally fully blind and naïve to SSD, performed the same experiment (see Fig. 2). Their overall performance was 61.3% ± 10.1, significantly lower than that of the blind (p < 0.0006). Interestingly, the sighted performed significantly above chance level (p < 0.0006), demonstrating that some aspects of the visual-to-auditory transformation and of ‘visual’ parsing using the device are intuitive.

However, when looking specifically at the stimuli of interest, i.e. the 3D shapes and the 2 overlapping shapes, the naïve sighted controls’ performance was much lower and did not differ significantly from chance (p > 0.05): 40% ± 8.6 correct for 2-overlapping line-drawing shapes, 37.1 ± 26.1% for 2 overlapping transparent shapes, and 51.4% ± 22.7 for 3D shapes. All scores were significantly lower than those of the blind (p = 0.0125, p = 0.0135 and p = 0.003, respectively). Thus, the blind participants’ success was not based on the auditory input alone but rather required visual interpretation.

**Figure 3:**
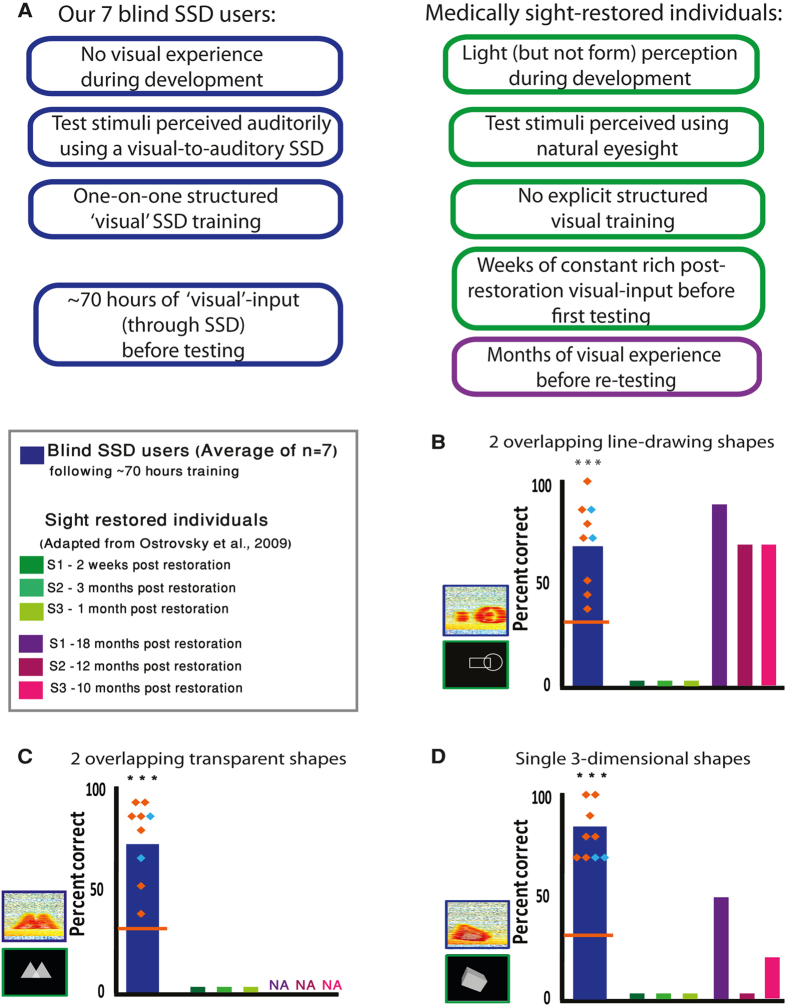
Comparison of ‘visual’ parsing abilities of the congenitally blind SSD-users and medically sight-restored individuals. (**A**) Main differences between the two groups. (**B**–**D**) Comparison of the performance of our group of blind SSD-users (who performed the task using SSD) vs. that of 3 sight-restored individuals tested by Ostrovsky *et al.*^13^ short and long time post-surgery (performed the task visually). The average performance of the 7 congenitally fully blind SSD-users is depicted by blue bars, performance of individual congenitally fully blind SSD-users by orange diamonds, performance of individual SSD-users who had very limited visual experience are depicted by cyan diamonds. Each sight-restored individual is represented by a green bar (performance tested a short time post-surgery) and a purple/pink bar (performance tested a longer time post-surgery). *** denotes p < 0.0006. The SSD-users outperformed the sight-restored individuals in most aspects tested, both at the group level and at the single-subject level. Data on the sight-restored individuals were adapted with permission from Ostrovsky *et al.*, 2009^13^. (**B**) Identifying 2 overlapping line-drawing shapes as 2 distinct objects. (**C**) Identifying 2 overlapping filled transparent shapes as 2 distinct objects. (**D**) Identifying a 3-dimensional shape as a single entity.

The SSD-users’ success was further manifested when comparing their individual achievements (represented by orange diamonds in Figs 1 and 3) to those of the 3 sight-restored individuals described in the intriguing work by Ostrovsky and colleagues^13^ (see Fig. 3A for a comparison between the groups’ characteristics). These individuals were tested on comparable static visual parsing tasks twice: two weeks to three months post-restoration, and again (on some of the tasks) 10–18 months post-restoration to determine progress. The stimuli used in our experiment were similar in principle, but not completely identical (e.g. some of the shapes were different) to those used in the sight restoration study. Moreover, we conducted a 3-alternative forced-choice experiment, to enable statistical analysis of significance, whereas in the sight restoration study free responses were required. Nevertheless, although a fully direct comparison was impossible, comparing the two studies was relevant and instructive.

When tested weeks post-restoration, the sight-restored patients failed on the three comparable tasks; i.e., parsing two overlapping line drawing shapes, parsing two overlapping transparent shapes, and parsing 3D shapes. Thus, each of the 9 SSD-users outperformed them (Fig. 3B–D). At the second time-point the sight-restored were tested, 10-18 months post-surgery, some improvement was reported in parsing overlapping line-drawing shapes. One patient had nearly-perfect performance, and the other two also made strides. Nevertheless, as can be seen in Fig. 3B four of the SSD-users still outperformed the sight-restored and 2 SSD-users had comparable performance. Finally, when tested again on 3D-parsing, one of the sight-restored subjects still had 0% success, but the other two showed some improvement. In this case, all the individual SSD-users outperformed the sight-restored patients (see Fig. 3D). Parsing of two overlapping transparent shapes in the sight-restored was not tested at this time-point, so no comparison could be made.

## Discussion

The findings show that a key complex visual concept – ‘visual’ parsing– can be learned and implemented in adulthood using sound-represented visual images, without any visual experience (Fig. 1). After participation in our unique structured-training program, the congenitally blind SSD-users experienced success (at both the group level and the single-subject level) on various parsing tasks: they correctly perceived 2D overlapping shapes in different formats as distinct objects, and perceived 3D objects as single entities (Fig. 1C,D). When considering our results one must understand how far from being trivial, although often taken for granted by the normally-developed sighted, is for the blind to acquire functional vision and to perform ‘visual’ tasks. Thus, for some medically sight-restored individuals learning to interpret regained visual information, and to actually see, was not only slow and challenging (as discussed in the introduction), but was so difficult that they regressed to living in functional self-defined blindness^10^,^11^.

We further had the opportunity to compare (Fig. 3) the parsing abilities of our subjects to those reported for individuals who regained sight by cataract removal or refractive correction^13^, i.e. to compare between the two visual rehabilitation approaches. This was a unique opportunity, as both highly-trained congenitally blind SSD-users and medically sight-restored individuals are relatively rare groups which are not easily accessed. We found that the SSD-users acquired this skill faster; namely, they succeeded on the task after only ~70 training hours (only ~20 minutes on the specific tested tasks), whereas the sight-restored patients completely failed even following weeks of constant natural vision (i.e., a minimum of 210 waking hours for the patient who was tested the shortest time post-surgery) – and in some aspects performed better than the sight-restored even after ~1 year of eyesight. This is especially intriguing when considering the complete lack of visual experience in 7 of our subjects (while the medically-restored patients had some light-perception throughout their life as these procedures require functioning photoreceptors). This finding has also strong relevance to any other means of sight-restoration since cataract removal represents the best case scenario in terms of the resulting resolution.

Finally, we showed in a control experiment with naïve sighted individuals (Fig. 2) that some (simple) aspects of the visual-to-auditory transformation and of ‘visual’ parsing using the device are intuitive. Moreover, the findings demonstrate that not only were the blind SSD-users better than the sight-restored, their abilities following training were also better than the intuitive understanding of the sighted individuals who had normal visual experience during development and could base their judgment on extensive visual knowledge.

Our results have both theoretical and practical implications, which will be discussed below.

On a theoretical level, the findings suggest that with adequate training and technologies, high-order visual **concepts** can be learned in adulthood using an out-of-the-box approach, and that complete lack of visual experience during the relatively narrow critical period window^32^,^33^ can be somewhat compensated for.

Because the visual-to-auditory transformation is not associative but rather preserves the visual spatial layout, because the task could not be performed based on auditory processing alone (Fig. 2) and because all the experimental stimuli were novel to the subjects, their success reflects the implementation of visual principles and a generalized learning of the tested skills.

Importantly, we do not claim that our subjects’ ‘visual’ abilities necessarily imply that they generated holistic 3D mental ‘visual’ representations. However, even if such a representation was not created, and the task was performed based on more local features in the image and/or on low level cues (probably at the level of a 2.5-dimension sketch as suggest by Marr^34^), and by using different strategies than normally sighted individuals, the results are still very encouraging both theoretically and in terms of rehabilitation practicability. They suggest that: 1) the information conveyed through the vOICe suffices to perform complex visual tasks; 2) various execution techniques can be learned such that visual capacities can be recovered in a top-down manner (based on feedback information from higher-order areas, previous experience and cognitive processing, all mediated through abundant backwards connectivity) even when bottom-up pathways are massively impaired (and will remain so even after an invasive intervention).

On a practical level, this is the first time that ‘visual’ parsing abilities using SSD were directly tested. This ability is necessary for using SSDs in everyday life since proper parsing of a visual scene into distinct whole objects is an initial step in recognizing them. Therefore, the participants’ success is very encouraging with regard to the potential of SSDs to aid the blind, providing them with otherwise unavailable visual information and capacities. SSDs may be especially beneficial for a specific sub-group of the blind who, due to their etiology, cannot undergo invasive restoration procedures (i.e. all congenitally fully blind individuals, and late blind individuals who have non-functioning components in the visual pathway between the operated areas and the brain), but will also be extremely helpful for the entire blind population since the vast majority resides in poor developing countries and have scant access to medical treatment (WHO fact sheet N282 2013).

Nevertheless, SSDs also have disadvantages^35^. These include the absence of subjective visual qualia^36^ (though see^37^), a need for organized structured-training, possible interference with environmental auditory inputs, and less automatic, more cognitively demanding perception. Visual-to-auditory SSDs are also slow compared to natural vision (e.g. ~7 seconds on average in the current experiment; though see also the relatively slow reaction time in a sight-restored individual^38^ and even slower times in retinal prosthesis implantees on similar or much easier tasks^5^). This is partially an integral component of the transformation algorithm which, in the case of the vOICe, displays the image sequentially. These disadvantages may (in addition to psychological and social factors) account for the fact that no SSD has been widely adopted by the blind to date.

This said, based on the behavioral achievements reported by us and by others^25^,^26^,^27^,^28^,^29^,^30^, together with the growing implementation of adequate training procedures (e.g. an online training that will help to expand SSD usage and training from the lab to the field^39^) and improvement in SSD technology (generating more user-friendly devices and upgrading their capabilities), SSDs have great promise for visual rehabilitation as standalone daily aids^35^.

**Figure 4:**
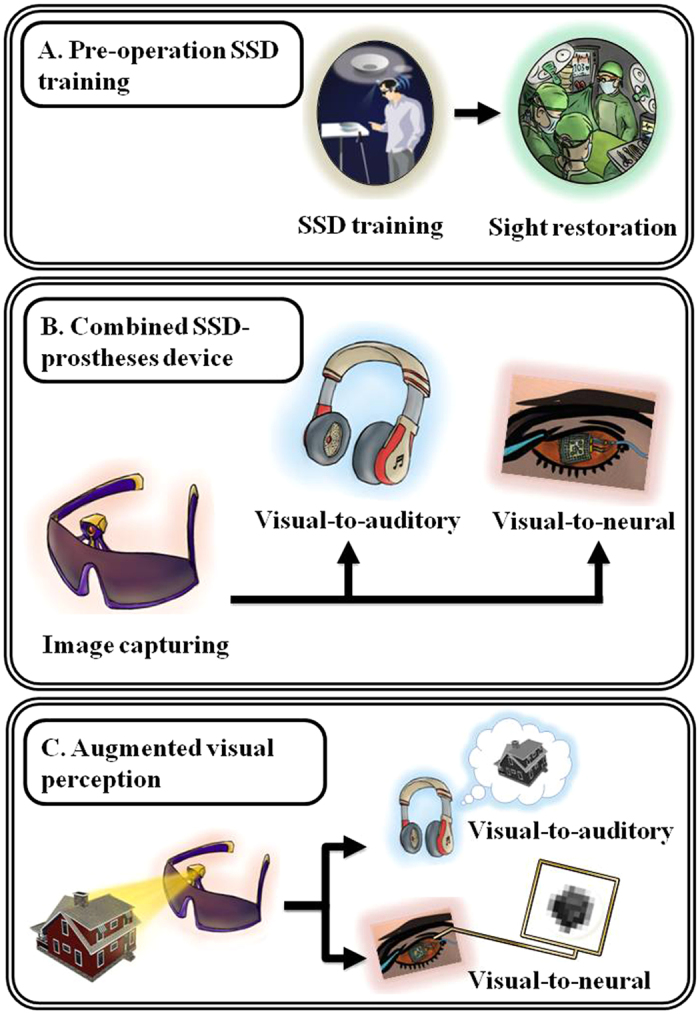
Combining SSDs with invasive means of sight-restoration to enhance visual abilities. (**A**) Using SSDs to visually train individuals before invasive sight restoration to familiarize them with visually-unique features, and to strengthen the cortical visual networks. (**B**) Combining SSDs with visual prostheses as a neuro-rehabilitative post-operation aid. The system includes: 1) A camera consistently capturing images; 2) A processing unit which converts the visual information into: (i) an auditory sensory-substitution representation and (ii) neural stimulation conveyed by the visual-prosthesis electrodes. In such a device, the prostheses provide vivid visual qualia and the SSD provide explanatory input to the visual signal from the prosthesis. The dual synchronous visual information is expected to speed up rehabilitation. (**C**) Using SSDs to provide input beyond the maximal capabilities of the prosthesis. Thus, the technical resolution of ‘the vOICe’ SSD stimulation can be up to two orders of magnitude higher than that of currently available prostheses^4^. Therefore, while the information from the prosthesis might not suffice for various visual tasks such as determining the shape and vantage point of the house, the additional SSD-input would enhance perception.

Furthermore, we suggest that SSDs can be complementarily and synergistically combined with invasive sight-restoration procedures, taking into consideration the advantages and disadvantages of each approach. Thus, SSDs can be used before invasive sight restoration procedures (see Fig. 4A), to familiarize the operated individual with unique visual features in order to ease rehabilitation. For instance, blind individuals might benefit from learning and practicing before surgery concepts like visual parsing which can be quickly learned with SSD (Figs 1 and 3), but were impaired following invasive procedures.

Moreover, a growing body of evidence shows that the ‘visual’ cortex of the blind follows the original functional organization and task specialization of the sighted visual cortex, and that SSD-‘vision’ recruits largely the same neural networks engaged by natural vision^29^,^40^,^41^,^42^,^43^,^44^ (reviewed in^45^,^46^). Therefore, prior SSD training may be also used to induce adult plasticity and strengthen the visual networks, thus supporting sight restoration efforts^47^.

Additionally, when the invasive procedure involves a visual prosthesis, a combined post-operation aid can be used (See Fig. 4B), delivering the visual information simultaneously through the prostheses electrodes (providing vivid visual qualia) and through SSD (providing explanatory input to the visual signal). Based on our demonstration (Fig. 3B–D) that the same visual task is learned faster by SSD-users than by sight-restored individuals, the dual, synchronous “visual” information should speed up rehabilitation.

Finally, SSDs can be used to provide input beyond the maximal capabilities of the prosthesis (Fig. 4C). Thus, the technical resolution of the vOICe SSD^31^ can be up to two orders of magnitude higher than that of current retinal prostheses^4^; and the functional ‘visual’ acuity of blind vOICe-users was shown to exceed the acuity reported with any visual rehabilitation approach^48^. Therefore, the information from the prosthesis might not suffice for various visual tasks, which could be relatively easily performed using SSDs (see the demonstration in Fig. 4C). Thus, an individual will probably be able to recognize the typical configuration of a face using the prosthesis, but in order to recognize facial expressions the SSD will have to be turned on. Complementary color and depth information, which are currently not conveyed through prostheses, can also be conveyed through recently developed SSDs^30^,^49^.

Taken together, SSDs has a great rehabilitative potential as standalone assistive aids or combined (pre/post-surgery) with invasive sight restoration techniques.

One interesting question, which is beyond the scope of this article, is why the SSD-users, who received the visual information through their ears, performed better and required less experience than the sight-restored individuals, who received the information in the natural way. One speculative explanation is that since the initial processing of the SSD-delivered information is likely to be carried out by the auditory system (e.g. identifying the sound frequency and timing), SSD ‘vision’ benefits from the superior auditory skills of the blind^50^, their greater reliance on audition in daily life and their richer auditory (vs. visual) experience. However, critically, none of the tasks reported here could be performed based on the auditory input alone, but rather required ‘visual’-specific processing (see Fig. 2).

Another not mutually exclusive explanation, which we believe played a central role in the achievements reported here, is the specific structured-training approach we used, during which the foundations of vision were gradually and explicitly taught, and various ‘visual’ tasks were intensively practiced. We stress that all (invasive or non-invasive) visual rehabilitation approaches should be accompanied by structured-training (see also^35^.) Training is important not only to the early blind but also to late blind individuals trying to cope with an atypical and degraded input such as that arriving from SSDs or visual prostheses (which is very different than natural stimulation of the neurons during eyesight). The importance of training was demonstrated for instance by showing that while sighted individuals were able to spontaneously extract SSD-conveyed pictorial depth cues and use them for depth estimation, early blind individuals were able to do so only following a training session in which they experienced various aspects of ‘visual’ depth^27^. Additionally, brain imaging studies have shown that SSD training strengthened the functional connectivity between the auditory cortex and task related ‘visual’ areas^42^; and that SSD-induced occipital cortex activation was stronger following training^51^. Even in the easier (and unilateral) case of visual impairment, amblyopia, a combined treatment which includes structured visual training was shown to trigger adult plasticity and greatly improve visual perceptual outcomes^52^.

Regardless of explanation, our results clearly show that the absence of visual experience should not limit the acquisition of ‘visual’ parsing - a critical high-level aspect of vision. Most probably, provided proper training, the ability of the blind to learn visual-unique concepts using out-of-the-box methods also applies to at least some other functions, such as size constancy. Future studies should examine this, as well as whether the action-perception loop can be closed using SSDs and/or other visual rehabilitation approaches^53^. Additionally, the practical contribution of SSDs as a means for ‘visual’ training before/after sight restoration and whether they indeed help overcome the serious deficits observed in practical visual perception after sight is regained still need thorough evaluation in future clinical trials. Finally, we plan to use the training program we developed to train also medically sight-restored patients who do not use SSDs, to test whether abilities can improve in a similar manner when eyesight alone is used in the training process.

## Methods

### Participants

Nine blind individuals (see Table 1 for full details) participated in the experiment. Seven were congenitally fully blind, one (FN) was congenitally blind but had faint light perception, and the remaining participant (HBH) had congenital blindness in her left eye and lost sight in the right eye at the age of 1 year. Subjects’ ages ranged from 21 to 53, all had normal hearing (except PH, who had slightly impaired hearing in her right ear), and had no neurological or psychiatric conditions. None of the participants had any experience with SSD prior to training. Additional seven sighted individuals, matched in gender and age to the seven congenitally and fully blind participants (average age: 33, range: 20–52) and totally naïve to SSDs (unfamiliar with the visual-to-auditory transformation algorithm), participated in the experiment as a control group. The Hebrew University’s ethics committee for research involving human subjects approved the experiments and written informed consent was obtained from each participant. All methods were carried out in accordance with the approved guidelines.

### “The vOICe” visual-to-auditory sensory substitution device

The vOICe SSD^31^ converts images into sounds preserving visual detail at a high resolution (up to 25,344 pixels, the resolution used here) using a pre-determined algorithm (see Supp. Fig. 1B for full details), thus enabling “seeing with sound” for highly trained users.

### Structured-training procedure

Blind participants were enrolled in a novel unique training program in which they learned how to extract and interpret visual information using the vOICe SSD. Each participant was trained for several months in a 2-hour weekly training session by a single trainer on a one-to-one basis. The training duration (71.2 hours on average) and progress rate varied across participants and was determined by personal achievements and difficulties as well as other time constraints.

The program was composed of two main stages. During the structured 2D training, participants learned to extract high detail 2D visual information from still (static) images. During each training trial, participants heard a soundscape and had to describe the image as accurately as possible and to recognize what they ‘saw’. Occasionally, mostly in the first few training sessions, the participants were asked to draw the image they ‘saw’ (by engraving, thus making the image tangible). This requirement forced them to reach definite conclusions as to how they imagined the image, and enabled the trainer to completely assess their ‘visual’ perception. In cases when the participants failed to perfectly describe the image, had mistaken or missed some details, they were asked guiding questions by the trainer, who also directed them as to the processing strategy they could use to interpret the sounds. Thus, participants were instructed to attend to various properties of the sound (e.g. its duration, whether the sound’s frequency is constant or changing, etc.). Then, they were encouraged to think what shape (or combination of shapes, creating a complex image) could be represented by these specific properties. Additional useful hints, such as the relative size of an object compared to a known object (e.g. the participant’s hand), were discussed. This active technique enabled the participants to better understand how to avoid mistakes in the future and which questions they should ask themselves to correctly interpret the soundscape. This prepared them for future independent use of the vOICe, without the trainer’s guidance. In addition to the verbal description of the sound and the image it represented, we provided the blind subjects with tangible image feedback, identical to those they “saw” using the vOICe, which provided further understanding of the image (see Supp. Fig. 1E).

Special emphasis was given to the features characterizing the object category. For example, in the body posture category participants were encouraged to mirror the posture presented, in the face category they were instructed how to identify features that characterize a face in general and features that differentiate faces (e.g. hair length, eye shape and position), and in the house category they were encouraged to identify the general structure of a building, as well as specific features such as number of floors, number and location of windows and the shape of the roof.

In the second training stage, participants practiced dynamic active ‘vision’ in real environments using a mobile setup of the vOICe (Supp. Fig. 1C). The difficulty of tasks practiced was gradual, starting with localizing and reaching for simple objects placed on a homogenous background, through “eye”-hand coordination tasks and finally distance estimation of objects, corridor navigation and obstacle avoidance. After these demonstrations of general principles, the second stage was not structured, and varied as a function of participants, such that every blind user was trained for the specific tasks that coincided with his/her specific needs and interests.

Importantly, in both training stages participants were demonstrated and taught more general visual perception principles that they were unfamiliar with such as variations in object size at different distances and the transparency of objects. These complex visual concepts were first explicitly explained (e.g. “if one object occludes another one, than the first must be closer”) and then were directly practiced to enable implementation of the acquired knowledge.

### ‘Visual’ parsing test

General experimental design: vOICe soundscapes of image stimuli were played in a fixed pseudo-randomized order, at a scanning rate of 2 seconds per image, using Presentation software (Neurobehavioral Systems, CA, USA). Participants indicated their answer using the keyboard. Each sound was played until the subject responded, and the next stimulus was presented only after the subject pressed the space key. Answers and reaction times were recorded for each trial. No feedback was given to the participants during the experiment. All stimuli in the experiment were novel, and were not presented to the subjects in any previous training session, thus requiring generalization of the learned skills.

Experimental stimuli: The methodology and stimuli were based on those used by Ostrovsky and colleagues^13^ (“Tests of Static Visual Parsing” section), who assessed visual parsing in medically sight-restored individuals. Image stimuli consisted of 1, 2 non-overlapping, 2 overlapping or 3 non-overlapping 2D shapes or a single 3D shape, and subjects had to indicate whether each stimulus contained 1, 2 or 3 shapes. The 2D shapes were a circle, square, rectangle, triangle and pentagon. The shapes were presented in one of three formats: line-drawings, filled opaque shapes (with different luminance levels for different shapes within an image) or filled transparent shapes (same luminance level for all shapes within a single image). The 3D objects were filled opaque shapes corresponding to the 2D shapes (e.g. a cube instead of a square). The stimuli of most interest were the 2-overlapping shapes and 3D shapes. The other stimuli were used to control for the subjects’ general ability to identify the number of distinct objects and to eliminate any potential psychological bias if most stimuli had contained the same number of objects. However, in order to decrease the number of stimuli in the experiment so that subjects would remain focused and attentive, there were fewer repetitions of the control stimuli than the stimuli of interest. Specifically, the experiment included a total of 95 stimuli (divided into two runs): 45 images with 2 overlapping shapes (15 images per shape format), 15 images with 2 non-overlapping shapes (5 stimuli per shape format), 15 images with 3 non-overlapping shapes (5 stimuli per format), 10 images with a single shape (5 in an outline format and 5 in a full solid shape format) and 10 stimuli with a single 3D shape. The shapes’ locations varied randomly between the images, thus the timing of objects in the soundscapes could not have been informative about their number. See Fig. 1B for examples of the different stimuli and their auditory representation.

Blind participants were briefly trained (~20 minutes; see Fig. 1A) for the specific task before the experiment to familiarize them with the visual principles of object occlusion, transparency, segmentation and overlap. During training, one stimulus of each type (i.e. a 3D shape, 2 overlapping filled opaque shapes, etc.) was presented using different shapes than those used in the experiment (a trapezoid, a rhombus, a cylinder). Sighted controls were not trained and remained naïve to the visual-to-auditory transformation algorithm.

### Statistical analysis

Average percent correct was calculated and a Wilcoxon rank sum test was used to test for significance (relative to chance level, which was 1/3, as there were 3 possible answers, or between blind and sighted groups). A Bonferroni correction was used to account for multiple comparisons. Thus, we divided the target α = 0.05 value by the number of statistical comparisons performed, which yielded p < 0.00625 as the threshold for significance. Additionally, the d’ sensitivity measure was calculated for the main results.

## Additional Information

**How to cite this article**: Reich, L. and Amedi, A. ‘Visual’ parsing can be taught quickly without visual experience during critical periods. *Sci. Rep.* **5**, 15359; doi: 10.1038/srep15359 (2015).

## Supplementary Material

Supplementary Information

## Figures and Tables

**Table 1:** Blind Participant Demographics.

Subject	Age(years)&gender	Cause of blindness	Lightperception	Age ofblindnessonset(years)	Musicalexperience	Prior SSDexperience	Braillereading
**EQ**	34 F	ROP	None	0	No	No	Yes
**FM**	28 F	Microphthalmia	None	0	No	No	Yes
**FN**	30 F	LCA	Faint	0	No	No	Yes
**HBH**	22 F	Microphthalmia, Retinal detachment	None	1	No	No	Yes
**IS**	29 F	ROP	None	0	Works as a music therapist	No	Yes
**PC**	36 M	ROP	None	0	No	No	Yes
**PH**	37 F	Congenital rubella	None	0	played musical instruments for ~7 years	No	Yes
**TT**	53 M	ROP	None	0	No	No	Yes
**UM**	21 F	ROP	None	0	No	No	Yes

ROP: Retinopathy of prematurity; LCA: Leber congenital amaurosis.
