# Supplementary material for: 'Visual’ parsing can be taught quickly without visual experience during critical periods
Source: Sci Rep. 2015 Oct 20;5:15359. doi: 10.1038/srep15359 (PMC4611203; doi:10.1038/srep15359)
Supplement: Supplementary Information [file srep15359-s1.pdf]

'Visual' parsing can be taught quickly without visual experience during critical periods

Lior Reich[1] and Amir Amedi[1,2,*]

[1]Department of Medical Neurobiology, The Institute for Medical Research Israel-Canada, Faculty of Medicine, The Hebrew University of Jerusalem, Jerusalem 91220, Israel.

[2]The Edmond and Lily Safra Center for Brain Sciences (ELSC), The Hebrew University of Jerusalem, Jerusalem 91220, Israel.

[*]Correspondence should be addressed to AA (amir.amedi@ekmd.huji.ac.il)

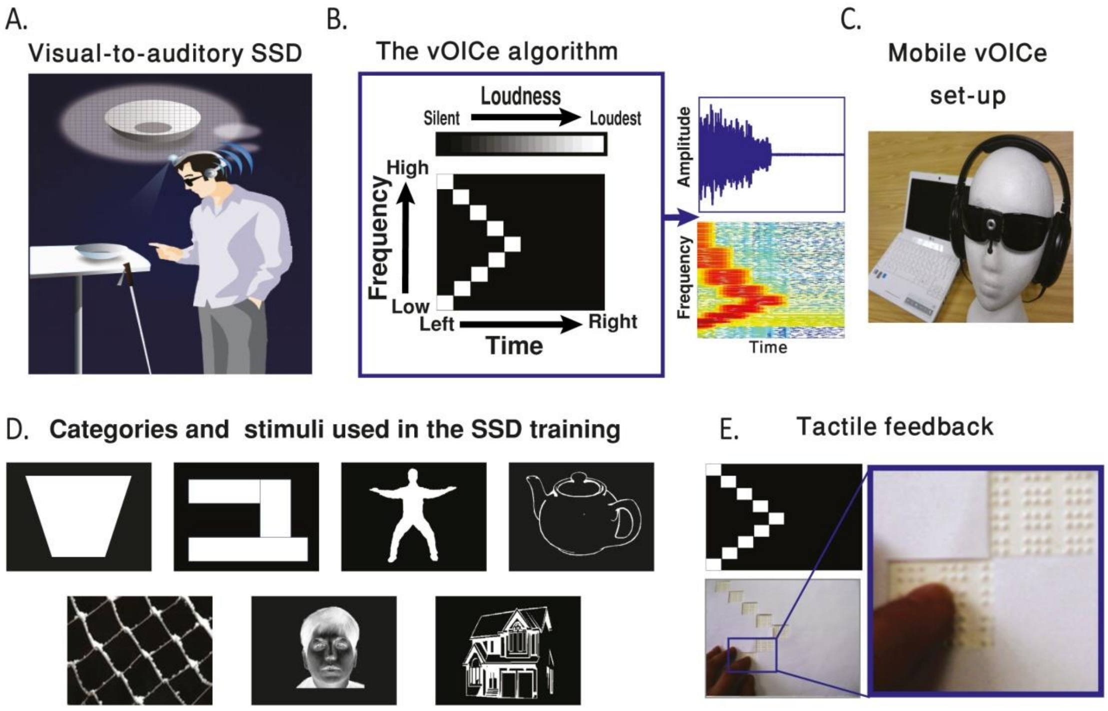

Supp. Fig. 1: Visual-to-Auditory sensory-substitution and training procedure. A. Visual-to-auditory SSDs convey visual information to the blind using sounds. **B.** The vOICe SSD[31] transformation algorithm: each image is scanned from left to right, such that time and stereo panning constitute the horizontal axis in the auditory representation ('soundscape'), tone frequency makes up the vertical axis, and loudness corresponds to pixel brightness. As can be seen in the spectrogram to the right, the soundscape preserves the visual shape. **C**. For mobile use of the device in everyday settings, users wear a lightweight inexpensive miniature video camera (worn on eyeglasses) connected to a computer/smartphone and earphones. **D.** Participants were taught how to extract and interpret visual information using the vOICe. During the structured 2-dimensional training stage, hundreds of images were introduced in a controlled order with growing complexity. Images were grouped into functionally relevant categories: geometric shapes, Hebrew letters and digital numbers, body postures, everyday objects, textures, faces and houses. Special emphasis was given to the features characterizing the different object categories.

E. Tangible images (bottom part), identical to the images presented using the vOICe (upper part), provided feedback to the blind during training**.**

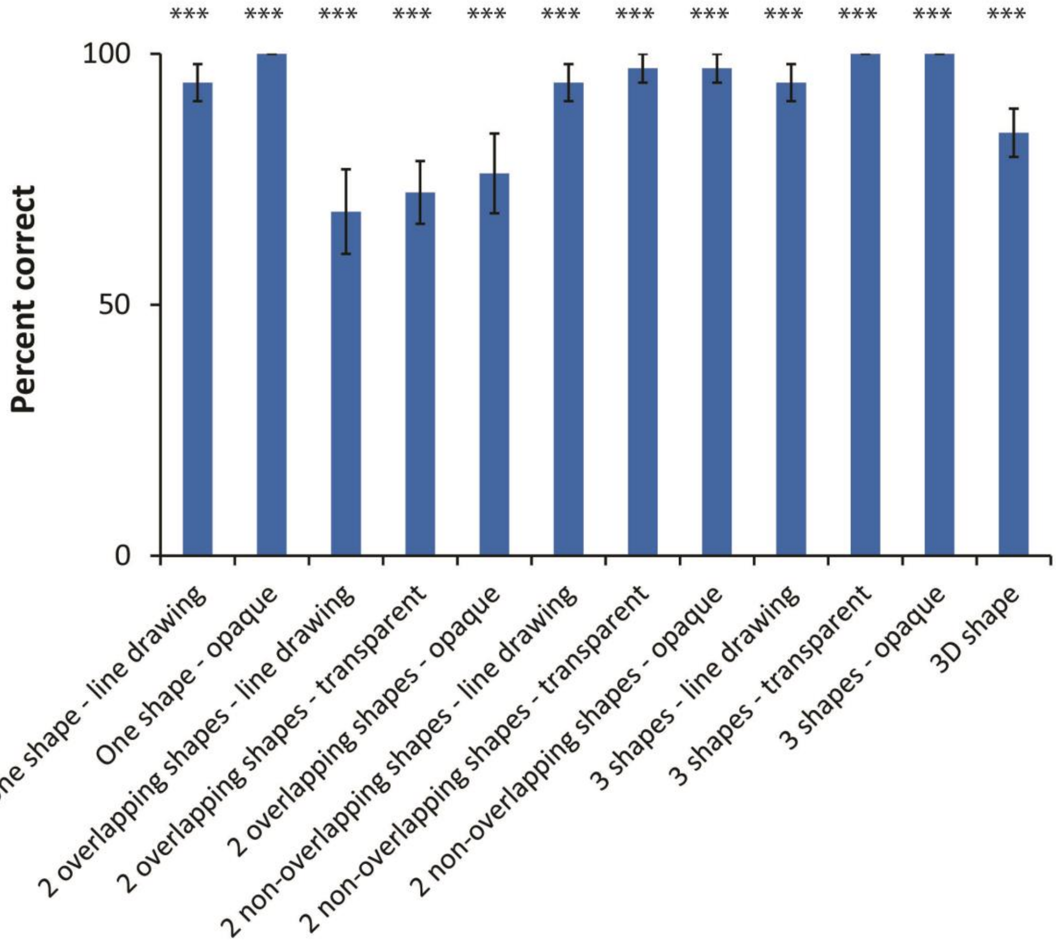

Supp. Fig. 2: Success rate of the SSD-users in each stimuli type tested. Average results for the group of 7 congenitally fully blind SSD users. Error bars represent standard error of the mean. *** denotes $p < 0.0006$.

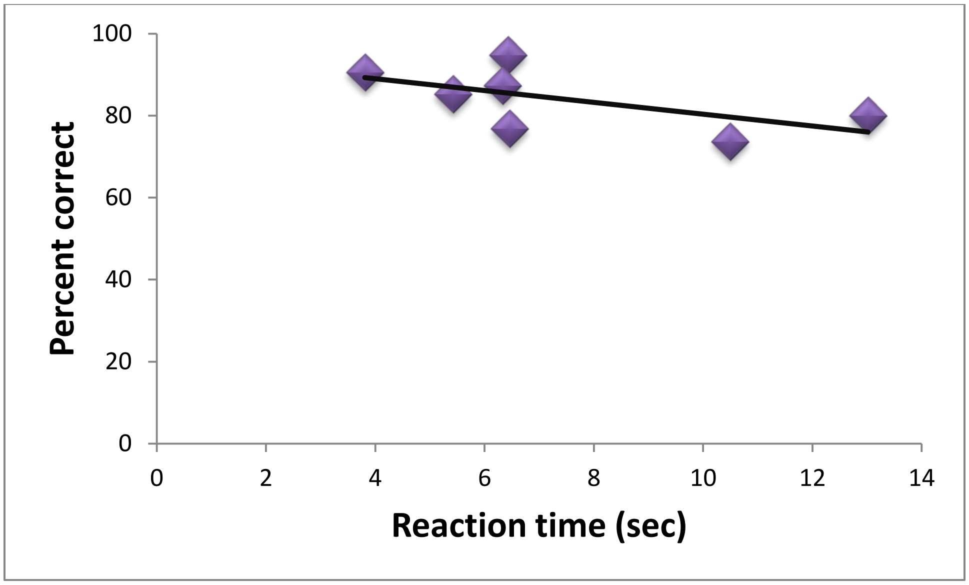

Supp. Fig. 3: No correlation between participant's performance and reaction time. The percent correct of each of the 7 congenitally fully blind participants was plotted against their average reaction time. No significant correlation was found (r2= 0.366).

	One shape - line drawing			One shape opaque			One 3D shape		
	"1"	"2"	"3"	"1"	"2"	"3"	"1"	"2"	"3"
EQ	4	1	0	5	0	0	7	3	0
FM	5	0	0	5	0	0	9	1	0
IS	5	0	0	5	0	0	8	2	0
PC	4	1	0	5	0	0	10	0	0
PH	5	0	0	5	0	0	7	3	0
TT	5	0	0	5	0	0	10	0	0
UM	5	0	0	5	0	0	8	2	0
Total	33	2	0	35	0	0	59	11	0

	2 overlapping shapes - line drawing			2 overlapping shapes - transparent			2 overlapping shapes - opaque		
	"1"	"2"	"3"	"1"	"2"	"3"	"1"	"2"	"3"
EQ	0	13	2	0	14	1	0	14	1
FM	7	8	0	7	8	0	7	8	0
IS	0	15	0	1	14	0	1	14	0
PC	7	5	3	1	11	3	1	13	1
PH	0	12	3	0	10	5	1	12	2
TT	8	7	0	7	8	0	8	6	1
UM	1	11	3	1	12	2	0	13	2
Total	23	71	11	17	77	11	18	80	7

	2 non-overlapping shapes - line drawing			2 non-overlapping shapes - transparent			2 non-overlapping shapes - opaque		
	"1"	"2"	"3"	"1"	"2"	"3"	"1"	"2"	"3"
EQ	0	4	1	0	5	0	0	5	0
FM	0	5	0	0	5	0	0	5	0
IS	0	4	1	0	5	0	0	5	0
PC	0	5	0	1	4	0	1	4	0
PH	0	5	0	0	5	0	0	5	0
TT	0	5	0	0	5	0	0	5	0
UM	0	5	0	0	5	0	0	5	0
Total	0	33	2	1	34	0	1	34	0

	3 shapes - line drawing			3 shapes - transparent			3 shapes -opaque		
	"1"	"2"	"3"	"1"	"2"	"3"	"1"	"2"	"3"
EQ	0	0	5	0	0	5	0	0	5
FM	0	0	5	0	0	5	0	0	5
IS	0	0	5	0	0	5	0	0	5
PC	0	1	4	0	0	5	0	0	5
PH	0	0	5	0	0	5	0	0	5
TT	0	1	4	0	0	5	0	0	5
UM	0	0	5	0	0	5	0	0	5
Total	0	2	33	0	0	35	0	0	35

Supp. Table 1: Response frequencies. Each row represents a different congenitally and fully blind participant. Columns indicate the number of times each response (“1”, “2” or “3”) was given for the different types of stimuli. The correct answer is marked in blue.
